# Perioperative care in pediatric OTC deficiency and goldenhar syndrome: a case report

**DOI:** 10.3389/fped.2025.1501423

**Published:** 2025-05-16

**Authors:** Daniela Torres Salazar, Andres Felipe Beltran, Sergio Alzate-Ricaurte

**Affiliations:** ^1^ Department of Anesthesiology, Fundación Valle del Lili, Cali, Colombia; ^2^ School of Medicine, Universidad Icesi, Cali, Colombia; ^3^ Centro de Investigaciones Clínicas, Fundación Valle del Lili, Cali, Colombia

**Keywords:** perioperative care, goldenhar syndrome, ornithine transcarbamylase deficiency disease, acid-base imbalance, craniofacial abnormalities

## Abstract

Ornithine transcarbamylase deficiency (OTCD) is an X-linked disorder disrupting the urea cycle in 1 per 56,000 births. Goldenhar syndrome (GS), characterized by craniofacial and vertebral anomalies, is seen in 1 per 3,000–5,000 births. Understanding the pathophysiology and manifestations of both is paramount for developing a perioperative plan. This case report presents a 21-month-old infant with both OTCD and GS undergoing hemifacial malformation correction surgery, the first such report in medical literature. The patient initially presented with lethargy, somnolence, headache, and seizures due to acute liver failure and metabolic acidosis. Diagnosis of OTCD was confirmed through elevated ammonia levels and urine organic acids analysis. The surgical plan involved mandible reconstruction using an autologous costochondral graft. Preoperative management included a low-protein diet and sodium benzoate administration to control ammonia levels. Anesthesia induction and maintenance were carefully managed, with close monitoring of metabolic parameters. The surgery lasted 4 h, during which the patient required transfusion therapy due to easy bleeding. Postoperatively, the patient was monitored in the Pediatric Intensive Care Unit for 48 h before transfer to the general ward. The importance of meticulous preoperative assessment, airway planning, and vigilant intraoperative management in OTCD and GS is underscored. This case highlights the challenges in managing rare comorbidities and emphasizes the need for a multidisciplinary approach. The collaborative effort between specialties led to successful management of this complex case, providing valuable insights for future similar scenarios.

## 1 Introduction

Ornithine transcarbamylase deficiency (OTCD) is an X-linked disorder disrupting the urea cycle, causing hyperammonemia. It is one of the most common urea cycle disorders, affecting 1 in 56,000 live births, though milder forms may go undiagnosed (1). Goldenhar syndrome (GS), is a rare congenital condition with an incidence of 1 in 3,000–1 in 5,000 live births, exhibiting craniofacial and vertebral anomalies. Its exact etiology is unknown but likely involves disruptions of branchial arches during embryonic development (2). Both conditions are rare, and their co-occurrence is exceptionally uncommon.

Understanding the pathophysiology and clinical manifestations of OTCD and GS is crucial for developing a comprehensive perioperative plan. These patients often present with multisystem involvement including neurological deficits, metabolic derangements, and craniofacial anomalies, impacting anesthesia and postoperative recovery (2, 3). Perioperative and anesthetic management requires a tailored approach for optimal outcomes. This is the first case reporting coexistence of both conditions. Written consent was obtained from the minor's legal guardian for access to medical records, publication of the manuscript, and images. The final version was submitted to the Institutional Review Board and approved under Act 13 of 2024.

## 2 Case description

A 21-month-old patient, weighing 9.7 kg, was diagnosed with GS at 6 months. Recently, the patient developed lethargy, somnolence, intense headache, and seizures, secondary to acute liver failure and metabolic acidosis. One month into ambulatory workup, laboratories revealed an ammonium level of 190 µmol/L (reference: 16–60 µmol/L) and a lactate level of 9.0 mmol/L (reference: 0.5–2.2 mmol/L), prompting an urgent referral to our institution for further evaluation and management.

Upon admission, physical evaluation noted facial asymmetry, left mandibular hypoplasia, a retruded lower jaw, microtia, and a left preauricular appendage. Laboratories included elevated ammonia (180 µmol/L), prolonged prothrombin time (31.3 s), thromboplastin time (55.7 s), and abnormal liver enzymes (alanine transaminase 477 U/L, aspartate transaminase 157.5 U/L). Initial tests included serum amino acid (AA) analysis, liver biopsy, and urine organic acids analysis. Ultrasonography ruled out oculo-vertebro-cervical syndrome. Serum AA analysis showed elevated lysine and decreased citrulline and arginine, suggesting an inborn error of metabolism. Viral and autoimmune hepatitis were ruled out, but liver biopsy was inconclusive. Urine organic acids analysis showed elevated orotic acid and uracil excretion, indicative of OTCD.

**Figure 1:**
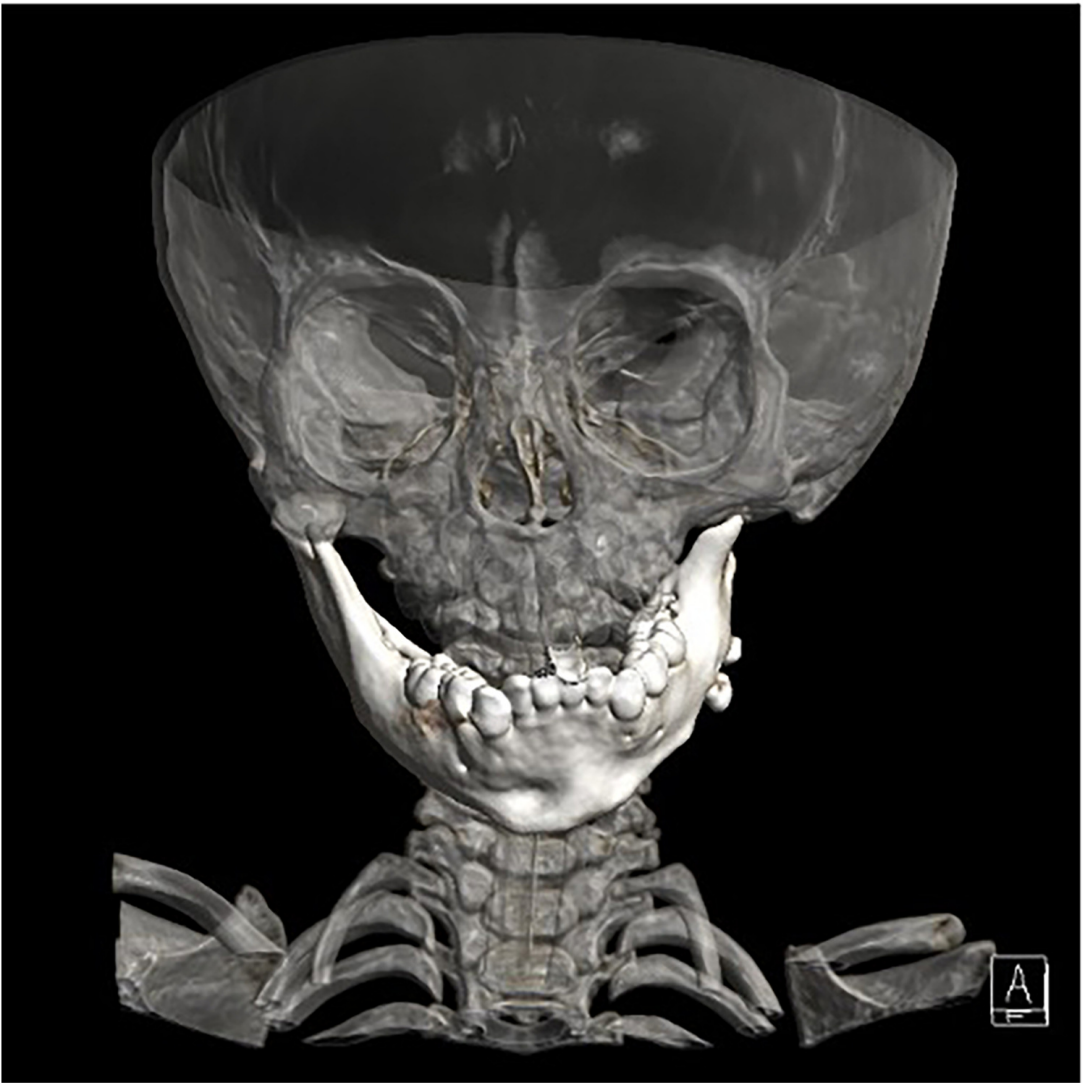
Imaging studies performed for surgical planning.

The final diagnosis was acute liver failure and metabolic acidosis secondary to OTCD. The complexity and rarity of this disorder posed a diagnostic challenge, especially given difficulties in accessing comprehensive genetic testing. Considering the social determinants, craniofacial surgery evaluation was recommended after clinical stabilization. Following stabilization, imaging studies led to a decision for mandible reconstruction using an autologous costochondral graft, to be harvested by the thoracic surgery department (Figure 1). Despite limited oral aperture, achieving a patent airway and ventilation was deemed possible based on previous procedures.

Preoperative laboratories showed elevated prothrombin time (24.8 s), thromboplastin time (50.8 s), alanine transaminase (144.5 U/L), aspartate transaminase (93 U/L), adequate hemoglobin (11.5 g/dl), decreased albumin (3.69 g/dl), and mildly elevated ammonia (60 µmol/L). Pediatric hepatology and nutrition teams initiated a low-protein diet. Three days before surgery, guided oral hydration and sodium benzoate (SB) administration decreased ammonia levels (43 µmol/L). Dextrose fluids were initiated at the start of fasting.

In the operating room, various difficult airway devices were prepared. Monitoring included pulse oximetry, capnography, electrocardiography, temperature, and non-invasive blood pressure. Anesthesia induction used sevoflurane (end tidal 2%), fentanyl (30 mcg), lidocaine (10 mg), and propofol (30 mg). After ensuring ventilation, rocuronium (10 mg) was administered, and airway control was achieved with a pediatric video laryngoscope and size #4 endotracheal tube. Following intubation, a gastric tube was inserted, and SB was administered. An arterial line was placed in the right radial artery, and a central venous catheter in the left internal jugular vein. Central venous pressure and invasive blood pressure were monitored, and a vesical catheter was placed. Anesthesia was maintained with sevoflurane (end tidal 2%), remifentanil infusion (0.1 mcg/kg/min), and dexmedetomidine infusion (0.5 mcg/kg/h) after an initial bolus of 10 mcg over one hour. Metabolic flow was maintained with 10% dextrose throughout surgery.

The patient exhibited easy bleeding during mandibular osteotomies. Transfusion therapy with 10 ml/kg of fresh frozen plasma and intravenous balanced solution was indicated. Arterial blood gas analysis after osteotomies showed a hemoglobin level of 10.9 g/dl, −5 base excess, and lactate level of 1.36 mmol/L. The procedure concluded after 4 h, with stable hemodynamics and minimal additional blood loss. The patient was transferred to the Pediatric Intensive Care Unit (PICU), extubated, and maintained stable hemodynamics with oxygen support. Postoperative ammonia levels were closely monitored. Therapy with SB and a protein-restricted diet continued. The patient remained in the PICU for 48 h without metabolic decompensation, then transferred to the general ward for an additional 14 days. The patient was discharged on day 31.

**Figure 2:**
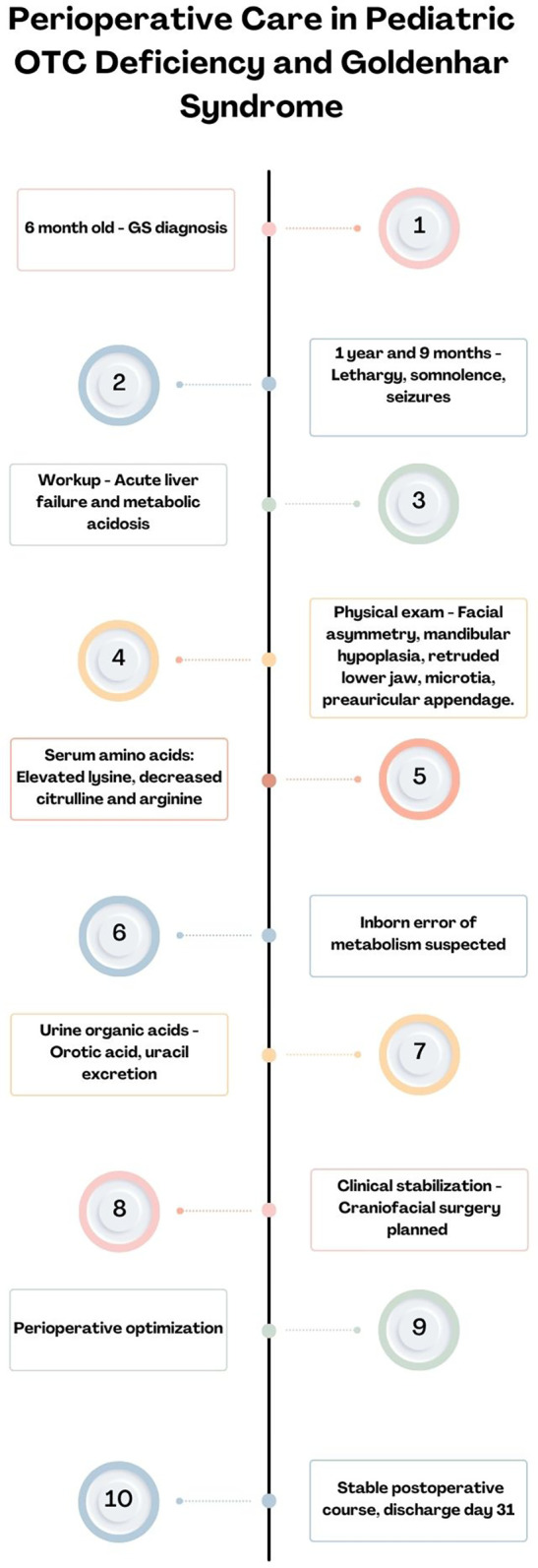
Timeline of case description events.

Postoperative follow-ups were not performed by the operating team due to social determinants limiting access. Over the past 2 years, the patient has been admitted for metabolic decompensations. However, during the last checkup 4 months ago with the nutrition team, the patient had no issues with feeding or mastication and a weight/stature relationship in the 78th percentile (Figure 2).

## 3 Discussion

This case is the first to report hemifacial malformation correction surgery in a patient with GS and OTCD. As GS is not typically comorbid with OTCD, this case highlights a rare coincidence of two unrelated pathologies occurring simultaneously.

Symptoms in OTCD range from severe neonatal hyperammonemia, coma, seizures, encephalopathy, and early death, to milder forms in partial deficiencies (4). Hyperammonemia results from the accumulation of glutamine and carbamoylphosphate and reduced synthesis of citrulline, arginine, and urea. Glutamate, aspartate, glutamine, and asparagine act as buffers to manage excess ammonia (5). Children with OTCD often require general anesthesia, increasing the risk of metabolic decompensation due to surgical stress (6). No studies have established best practices; however, maintaining hydration and therapies to reduce protein catabolism and enhance nitrogen excretion are recommended (7–9). These principles are supported by international guidelines, which emphasize early initiation of glucose infusions, protein restriction, and ammonia-scavenging therapy to prevent acute hyperammonemic crises (10).

To prevent acute decompensation, perioperative assessment should involve multidisciplinary collaboration, as illustrated in our case. Blood levels of ammonia and AAs should be closely monitored, and elective surgery postponed if there are signs of infection or metabolic decompensation. Patients are advised to follow low-protein diets, obtaining most of their calories from carbohydrates or lipids (7). The long-term dietary management strategies followed in this case are consistent with published consensus, which supports low protein intake, essential amino acid supplementation, and vigilant nutritional monitoring (10). Sodium benzoate (SB) is crucial for treating OTCD, as it provides an alternative pathway for nitrogen removal (11). SB and arginine should be administered the day before surgery and adjusted by ammonia levels. These actions align with guideline-based approaches to maintain metabolic stability during high-risk procedures (10).

Monitoring varies according to the procedure's nature. In our case, invasive monitoring was used due to a moderate bleeding risk and prolonged operating time. Difficult airway devices were available to account for craniofacial malformations. Volatile anesthetics have been reported for maintenance, and information on using propofol is unavailable (7, 12). Detailed descriptions on the use of locoregional anesthesia with bupivacaine in this context are unavailable (13). Ropivacaine has a safe pharmacological profile and selectivity, as it is metabolized by CYP1A2 in the liver. Lack of interaction with the urea cycle does not suggest a physiological basis to contraindicate its use (14). Bupivacaine was used for the infiltration of surgical wounds.

Focus should be on monitoring perioperative volume status to prevent dehydration and on potassium and glucose levels (7). During and after surgery, gas analysis, glucose levels, and plasma ammonia were measured for early detection of decompensation.

Perioperative hyperammonemia crises require prompt recognition and intervention to prevent neurological damage and mortality. In anesthetized patients, clinical signs may be absent; thus, proactive monitoring of serum ammonia levels is essential in at-risk individuals. Immediate management includes cessation of protein intake, optimization of caloric support to reverse catabolism, and administration of rescue nitrogen scavengers. These include sodium benzoate and sodium phenylbutyrate or phenylacetate, typically given as a 250 mg/kg loading dose over 90–120 min followed by a maintenance infusion. If plasma ammonia exceeds 500 µmol/L or fails to decrease within four hours of intensive therapy, dialysis should be initiated (15). Hemodialysis offers the most effective and rapid reduction in ammonia levels, followed by continuous venovenous hemodiafiltration (CVVHD) to maintain control. Coordination with a metabolic specialist and care in a pediatric ICU are essential for optimal outcomes (10).

Despite the moderate bleeding risk, we did not use tranexamic acid due to its association with hyperammonemia (8, 11). Instead, early use of fresh frozen plasma achieved adequate bleeding control during osteotomies, preventing unwanted volume loss.

The use of antiemetic prophylaxis for surgery in OTCD is debated. Some suggest that vomiting can indicate hyperammonemia and shouldn't be suppressed. However, we administered ondansetron due to its safety and efficacy in OTCD (16). This aligns with Orphanet consensus, which supports its use to minimize vomiting risk during scavenger boluses. We concluded that the effect of postoperative nausea and vomiting on recovery outweighs any benefits of vomiting as a clinical sign. To promptly evaluate neurological status and signs of decompensation, our objective was rapid extubation followed by postoperative monitoring in the PICU.

Long-term care for OTCD requires regular monitoring of ammonia levels to prevent recurrent subclinical hyperammonemia, which has been associated with progressive neurocognitive impairment. Nutritional management should ensure adequate energy intake while minimizing protein overload, often requiring individualized plans with essential amino acid supplementation. In patients with recurrent crises despite optimal medical management, liver transplantation remains a definitive therapeutic option and should be considered early in severe phenotypes (10).

A limitation of this report is the absence of genetic confirmation of OTCD, which remains the gold standard for diagnosis. Due to social and resource limitations, inpatient testing was not feasible at the time of admission; however, outpatient genetic evaluation was planned as part of the long-term management strategy. Additionally, long-term follow-up was not available due to significant social barriers to continuity of care. Nonetheless, it is important to emphasize that continued metabolic and developmental follow-up is standard of care for all patients with OTCD and remains strongly recommended in this case.

This case underscores the importance of meticulous preoperative assessment, careful airway planning, and vigilant intraoperative management in pediatric patients with complex conditions like OTCD and GS. Our patient did not experience excessive ammonia levels or metabolic decompensation. The collaborative effort between specialties and a well-coordinated anesthetic approach contributed to the successful management of this challenging case.

## Data Availability

The original contributions presented in the study are included in the article/Supplementary Material, further inquiries can be directed to the corresponding author.
